# Latent profiles of future care preparation among older adults with stroke and associations with filial responsibility expectations

**DOI:** 10.3389/fpubh.2026.1798813

**Published:** 2026-04-02

**Authors:** Lingli Li, Zefeng Bao, Qi Zheng, Jing Yang, Xuexin Liu, Rongrong Fan, Zehui Feng

**Affiliations:** 1 Sinopharm Dongfeng General Hospital, Hubei University of Medicine, Shiyan, Hubei, China; 2 School of Nursing, Hubei University of Medicine, Shiyan, Hubei, China

**Keywords:** older adults, older adults with stroke, filial responsibility expectations, future care preparation, latent profile analysis

## Abstract

**Aim:**

To identify latent categories of future care preparation in older adults with stroke and analyze their relation to filial responsibility expectations and influencing factors.

**Design:**

A cross-sectional, descriptive exploratory design with the STROBE reporting checklist was applied.

**Methods:**

Convenience sampling was employed to select 414 older adults with stroke from the neurology, geriatrics, and rehabilitation departments of a Grade A tertiary hospital in Hubei Province, China, between July and December 2025. Data were collected using a general information questionnaire, the Chinese version of the Preparedness for Future Care Needs Scale (PFCN-14), and the Filial Responsibility Expectations Scale (FRS). Latent profile analysis was conducted using the scores from the four dimensions of the PFCN-14 as manifest variables, and statistical analysis was performed using univariate analysis and multivariate logistic regression.

**Results:**

Latent profile analysis identified three potential categories of future care preparation: the low FCP-high avoidance group (17.8%), the moderate FCP-low planning group (45.2%), and the high FCP-moderate avoidance group (37.0%). Multivariate logistic regression analysis revealed that lower levels of filial responsibility expectations, younger age (60–79 years old), female gender, and close intergenerational relationships were significant influencing factors for patients’ classification into the high FCP-moderate avoidance group.

**Conclusion:**

This study found three future care preparation categories in older adults with stroke, showing differences in preparedness and avoidance. Formulating interventions requires considering each category’s physiological and psychosocial traits. Healthcare professionals should tailor strategies to boost patients’ future care preparation ability and willingness.

**Impact:**

Healthcare professionals should early identify patients in different preparedness categories, especially the low FCP-high avoidance group. Implement family-collaborative interventions, respecting patients’ wishes, to guide gradual future care preparation. Transform traditional filial piety into supportive resources to boost effectiveness and feasibility.

## 1 Introduction

Stroke is a severe cerebrovascular disease characterized by cerebrovascular neurological dysfunction, marked by high rates of disability, mortality, and recurrence (1). Globally, there are over 12.2 million new stroke cases annually, with stroke survivors facing a significant risk of recurrence—estimated at 11.1% within 1 year and reaching up to 39.7% over 12 years (2). In China, approximately 2 million new stroke cases occur each year, making it the country with the heaviest global burden of stroke (3). Population aging stands as the primary driver behind the escalating global burden of stroke. From 1990 to 2021, the number of stroke patients aged 60 and above surged from 20.1 million to 45.52 million, marking a 125% increase (4). With the rapid aging of the population and the persistently high prevalence rates of chronic diseases such as hypertension and their risk factors, it is expected that the incidence of stroke will further increase in the future (5). Age-related physical decline and long-term functional impairments resulting from stroke will generate substantial and ongoing care demands, further imposing potential pressures on patients themselves, their families, and society as a whole (6).

Future care preparation (FCP) refers to a series of preparatory activities undertaken by older adults in anticipation of requiring care from others due to chronic illnesses or health events in the future (7). Based on the proactive coping theory (8) and the theory of everyday planning (9), Sörensen et al. (10) conceptualized FCP as a process encompassing awareness or avoidance, information gathering, decision-making, and concrete planning. As a proactive form of long-term risk management, FCP enables older adults with stroke to proactively identify alternative caregivers or professional care institutions and adjust their care-coping strategies (11). This not only alleviates family caregiving pressure, reduces the risk of stroke recurrence, and delays the onset of disability, but also mitigates older adults’ sense of insecurity about the future, alleviates depression and anxiety, and enhances the alignment between their care preferences and the services they actually receive, achieving a win-win outcome for patients, families, healthcare systems, and society (12, 13). Due to the long-term impact of China’s family planning policy, the family structure is undergoing a “4-2-1” crisis (14). Families with older adults who have had a stroke often confront the dilemma of older adults caring for older adults. Therefore, focusing on enhancing the level of FCP among older adults with stroke is crucial for alleviating the burden of older adults care in the context of population aging, holding significant practical implications and social value.

For older adults with stroke with dual impairments in cognitive and physical functions, actively engaging in FCP not only helps maintain personal autonomy but also lays a crucial foundation for achieving successful aging. However, this group faces multiple difficulties in FCP. Firstly, due to their current poor health conditions, older adults with stroke often focus more on existing health issues and find it challenging to take into account future care needs, resulting in generally low levels of FCP (15). Secondly, stroke patients have more complex future care requirements compared to ordinary older adults because of characteristics such as physiological function decline and the coexistence of multiple diseases. In addition, the sudden disability caused by stroke itself, the unpredictable rehabilitation trajectory, and potential cognitive impairments may further undermine patients’ ability to plan for the future (16).

As aging progresses and self-care ability declines, coupled with a deficiency in their own planning ability, older adults with stroke often have higher expectations of their children’s filial piety responsibilities. Filial responsibility is defined as the social norms and cultural expectations surrounding the process by which adult children care for their aging parents (17). From the perspective of parents, this responsibility manifests specifically as expectations of filial piety from their children (18). Excessive reliance on adult children can hinder older adults with stroke from engaging in FCP. In a high-filial-piety cultural context, older patients commonly perceive that their children should fully shoulder caregiving responsibilities, thereby placing themselves in a passive position in care planning and weakening their intrinsic motivation to actively participate and make decisions (19). Meanwhile, filial norms may suppress open communication within families regarding caregiving issues. Out of a desire to maintain family harmony and avoid imposing psychological burdens on their children, older patients often shy away from discussing sensitive topics such as disability, death, and caregiving choices (20). Similarly, children may find it difficult to initiate relevant conversations for fear of being perceived as “unfilial” (21). This mutual silence results in unclear caregiving needs and misaligned preferences, impeding the formation of feasible care plans. Existing research has indicated that, due to traditional beliefs assigning the responsibility of long-term care to adult children, older patients in China exhibit notably insufficient preparation for future care (15), inadvertently excluding professional and socialized care support options. This not only exacerbates the burden of family caregiving but also leads to issues such as a diminished sense of control over life, depression, and anxiety (22).

Most existing studies on FCP employ a variable-centered approach to evaluate the overall level of preparation. Currently, there is a lack of research on the latent characteristics of FCP among older adults with stroke, particularly within the context of a high filial responsibility culture. Older adults with stroke exhibit individual differences in cognitive function, social support, and health status, which may lead to different patterns of FCP. Latent profile analysis (LPA), as a person-centered approach, can effectively uncover their heterogeneity. By analyzing individuals’ scores on observed variables, LPA determines the latent characteristics of individuals and their proportions within the population (23). Identifying the latent profiles and characteristics of FCP among older adults with stroke can, on the one hand, assist healthcare professionals in developing personalized intervention strategies for different patient subgroups and, on the other hand, enable early identification of patients with inadequate preparation and provide timely guidance. Exploring how expectations of filial responsibility influence different FCP profiles can offer a basis for culturally sensitive family interventions. These findings hold significant reference value for numerous regions worldwide that are experiencing low fertility rates and profound aging. They can help the growing population of older adults with stroke and their families break the silence, initiate communication, and make feasible FCP plans that align with patients’ wishes before the disease leads to complete disability, thereby avoiding family crises, improving patients’ quality of life in their later years, and promoting the rational utilization of social care resources. Therefore, this study aims to identify latent profiles of FCP among older adults with stroke and examine their association with filial responsibility expectations and other influencing factors.

## 2 Methods

### 2.1 Design

This study adopted a cross-sectional design in accordance with the STROBE guidelines for reporting observational studies (24). A convenience sampling method was used to recruit older adults with stroke who attended the neurology, geriatrics, and rehabilitation departments of a large public Grade A tertiary teaching hospital (with more than 1,500 beds) in Hubei Province, China, between July and December 2025.

### 2.2 Sampling and inclusion criteria

The inclusion criteria for patients in this study were as follows: (1) Age ≥60 years; (2) Meeting the diagnostic criteria outlined in the Chinese Guidelines for the Diagnosis and Treatment of Cerebral Hemorrhage (2019) (25), confirmed by head MRI/CT examination; (3) Patients with no further progression of neurological symptoms and signs and currently in a stable phase of the condition; (4) Providing informed consent and voluntarily participating in this survey. The exclusion criteria were as follows: (1) Patients with a documented diagnosis of cognitive impairment (e.g., dementia, severe cognitive deficits) based on medical records or the judgment of the attending physician; (2) Those with mental illness; (3) Those with hearing, visual, or verbal communication disorders; (4) Those with other major diseases, such as organ failure or malignant tumors; and (5) Those currently participating in other studies.

The sample size calculation formula for cross-sectional studies is given by $N={\left((Z_{\alpha /2}*\sigma )/\delta \right)}^{2}$ , where N represents the estimated sample size, $\;Z_{\alpha /2}$ is the value corresponding to the test level α,σ is the standard deviation, and *δ* is the allowable error. In this study, with a test level *α* = 0.05, $Z_{\alpha /2}$ = 1.96. A preliminary survey was conducted on 50 older adults with stroke to assess their FCP, revealing a mean item score of (2.94 ± 0.79) points. The standard deviation was taken as *σ* = 0.79. Based on a previous study (6), where the mean item score for FCP among older adults was 2.79 points, the allowable error was calculated as *δ* = 0.15. Using the formula, the estimated sample size was *N* = 107. Considering that 10% of questionnaires may be invalid or participants may be lost to follow-up during the survey process, resulting in a final minimum sample size of at least 119 older adults with stroke. LPA generally suggests a minimum sample size of 300–500 cases (26). Therefore, the sample size of 414 cases meets the requirement.

### 2.3 Data collection

This study was conducted with the approval of two neurology wards, one geriatrics ward, one rehabilitation ward, and the relevant hospital. During the on-site implementation of paper-based questionnaires, the researchers used identical instructions to explain the study’s purpose and questionnaire completion methods to all participants, obtaining the cooperation of older adults with stroke and securing their signed informed consent. Patients were asked to complete the questionnaires independently. For participants who encountered difficulties in filling out the questionnaires, the study adopted an approach where researchers posed questions and participants provided responses. Prior to the survey, each participant was formally informed of their rights in the study, including the right to withdraw. Standardized guidance was followed during the data entry process. After data collection, two individuals cross-checked and organized the data, excluding invalid questionnaires.

### 2.4 Measures

#### 2.4.1 Sociodemographic and clinical characteristics

The General Information Questionnaire was designed by the researchers of this study based on a review of relevant literature. The survey items encompassed age, sex, marital status, average monthly household income per capita, educational level, number of children, closeness to children, sex of children, place of residence, number of chronic diseases, and living arrangements.

#### 2.4.2 Preparation for Future Care Needs Scale-14

The Preparation for Future Care Needs (PFCN) scale was developed by Sörensen and Pinquart (27) in 2001 to assess the preparedness of older adults (aged >60 years) for future care needs. In 2022, Bai et al. (6) localized and adapted the PFCN-15 and PFCN-5 scales for use among older adults in Hong Kong, resulting in the Chinese version of the Future Care Needs Preparation Scale (PFCN-14). The PFCN-14 scale comprises four dimensions: awareness (4 items), avoidance (3 items), decision-making (3 items), and concrete planning (4 items), totaling 14 items. It employs a Likert 5-point rating scale, with “completely incorrect” scoring 1 point and “completely correct” scoring 5 points. The avoidance dimension is reverse-scored. The total score ranges from 14 to 70, with higher scores indicating greater preparedness for future care. The Cronbach’s *α* for the overall scale is 0.889, and for each dimension, it ranges from 0.749 to 0.928, suggesting good reliability of the scale.

#### 2.4.3 Filial Responsibility Scale

van der Pas et al. (28) revised the Filial Responsibility Scale (FRS) in 2005. This scale encompasses four dimensions: emotional expectations, instrumental expectations, contact expectations, and information expectations, totaling 16 items. The Chinese version of the FRS employs a Likert 5-point scoring method to assess older adults’ expectations of filial piety from their children, with scores ranging from 1 for “strongly disagree” to 5 for “strongly agree.” The total score ranges from 16 to 80, with higher scores indicating a higher level of filial piety expectations. The Cronbach’s *α* coefficient for this scale is 0.96 (29).

### 2.5 Data analysis

Data analysis was conducted using SPSS 27.0 and Mplus 8.3 software. For descriptive statistics, mean ± standard deviation was used to describe continuous data with a normal distribution, while frequency [percentage (%)] was employed to describe categorical variables. Latent profile analysis was performed using the scores from the four dimensions of the PFCN-14 scale as observed variables to identify potential types of FCP among older adults with stroke. Initially, a single-category model was hypothesized, followed by gradually increasing the number of categories. Parameters for each model were analyzed, and the optimal model was selected based on fit indices. The relevant fit indices included: (1) Akaike Information Criterion (AIC), Bayesian Information Criterion (BIC), and adjusted Bayesian Information Criterion (aBIC). Lower values of AIC, BIC, and aBIC indicated better model fit. (2) The Lo-Mendell-Rubin (LMR) test and bootstrap likelihood ratio test (BLRT) were used to compare the fit of the *k*-category model with that of the *k* − 1-category model. A significant *p*-value indicated that the *k*-category model was significantly superior to the *k* − 1-category model (30). (3) The Entropy index, with values closer to 1 indicating higher classification accuracy (31). The Chi-square test or Fisher’s exact probability method was used to compare differences in general data among various profiles. One-way ANOVA was conducted to explore differences in expectations of filial responsibility. The classification results from latent profile analysis served as the dependent variable, and statistically significant variables were selected as independent variables through inter-group comparisons. Multivariate logistic regression analysis was employed to identify factors influencing FCP among different latent categories of older adults with stroke. A *p*-value of less than 0.05 was considered statistically significant.

## 3 Results

### 3.1 Participants’ characteristics

This study ultimately included 414 participants (mean age: 71.62 ± 5.89 years), with a valid questionnaire response rate of 98.6% (6 participants were excluded from the initial 420 due to incomplete or invalid responses). The sample predominantly consisted of individuals aged 70–79 years (49.7%), males (67.1%), and married individuals (97.8%), with the majority having an educational level of primary school or below (44.4%). In terms of living arrangements, most participants lived with family members (80.9%), and 70.0% reported having a close relationship with their children. Detailed demographic characteristics of the participants are presented in Table 1.

**Table 1:** Differences in sociodemographic, disease-related characteristics and continuous variables among the latent profiles (*N* = 414).

Variables	Low FCP-high avoidance group (%)	Moderate FCP-low planning group (%)	High FCP-moderate avoidance group (%)	Total sample (%)	Statistical values	*p*
Age					26.315^a^	<0.001
60–69	15 (20.3)	76 (40.7)	74 (48.4)	165 (39.9)		
70–79	42 (56.7)	93 (49.7)	71 (46.4)	206 (49.7)		
≥80	17 (23.0)	18 (9.6%)	8 (5.2%)	43 (10.4%)		
Sex					9.370^a^	0.009
Men	56 (75.7)	133 (71.1)	89 (58.2)	278 (67.1)		
Women	18 (24.3)	54 (28.9)	64 (41.8)	136 (32.9)		
Marital status					2.245^b^	0.292
Married	72 (97.3)	185 (98.9)	148 (96.7)	405 (97.8)		
Others	2 (2.7)	2 (1.1)	5 (3.3)	9 (2.2)		
Monthly income					8.742^a^	0.068
<2000 RMB	31 (41.9)	62 (33.2)	48 (31.4)	141 (34.1)		
2000–5000 RMB	25 (33.8)	83 (44.3)	53 (34.6)	161 (38.9)		
>5000RMB	18 (24.3)	42 (22.5)	52 (34.0)	112 (27.1)		
Educational level					13.090^a^	0.042
Primary school/lower	39 (52.7)	87 (46.5)	58 (37.9)	184 (44.4)		
Middle school	20 (27.0)	52 (27.8)	40 (26.1)	112 (27.1)		
High school	10 (13.5)	35 (18.7)	29 (19.0)	74 (17.9)		
Junior college/higher	5 (6.8)	13 (7.0)	26 (17.0)	44 (10.6)		
Number of children					11.070^a^	0.026
≤1	10 (13.5)	28 (15.0)	37 (24.2)	75 (18.1)		
2-3	38 (51.4)	84 (44.9)	77 (50.3)	199 (48.1)		
≥4	26 (35.1)	75 (40.1)	39 (25.5)	140 (33.8)		
Closeness to children					43.937^a^	<0.001
Yes	36 (48.6)	119 (63.6)	135 (88.2)	290 (70.0)		
No	38 (51.4)	68 (36.4)	18 (11.8)	124 (30.0)		
Sex of children					37.279^a^	<0.001
Men	42 (56.8)	85 (45.5)	39 (25.5)	166 (40.1)		
Women	11 (14.8)	43 (23.0)	71 (46.4)	125 (30.2)		
Both	21 (28.4)	59 (31.5)	43 (28.1)	123 (29.7)		
Place of residence					12.548^a^	0.002
City	51 (68.9)	126 (67.4)	128 (83.7)	305 (73.7)		
Rural	23 (31.1)	61 (32.6)	25 (16.3)	109 (26.3)		
Living arrangements					9.807^a^	0.044
Living alone	8 (10.8)	24 (12.8)	9 (5.9)	41 (9.9)		
Living with family	57 (77.0)	153 (81.8)	125 (81.7)	335 (80.9)		
Others	9 (12.2)	10 (5.4)	19 (12.4)	38 (9.2)		
Number of chronic diseases					5.521^a^	0.238
1	21 (28.4)	64 (34.2)	58 (37.9)	143 (34.5)		
2	23 (31.1)	63 (33.7)	56 (36.6)	142 (34.3)		
≥3	30 (40.5)	60 (32.1)	39 (25.5)	129 (31.2)		
Filial responsibility expectations	64.97 ± 5.99	59.92 ± 7.27	55.46 ± 7.47	59.17 ± 7.88	46.173^c^	<0.001
Emotional	16.32 ± 2.48	14.80 ± 2.62	13.82 ± 3.03	—	20.824^c^	<0.001
Instrumental	16.38 ± 1.53	15.89 ± 2.55	15.22 ± 2.95	—	5.815^c^	0.003
Contact	16.11 ± 2.20	14.94 ± 2.58	13.47 ± 2.76	—	28.690^c^	<0.001
Information oriented	16.16 ± 2.51	14.27 ± 2.85	12.78 ± 3.11	—	35.072^c^	<0.001

a Chi-square test.

b Fisher’s exact probability.

c The one-way ANOVA.

### 3.2 LPA of participants

Based on the scores from the four dimensions of the PFCN-14, this study sequentially fitted models with one to five latent categories. The model fit indices are presented in Table 2. As the number of categories increased, the values of AIC, BIC, and aBIC gradually decreased, and the entropy values for all models were above 0.8. Except for Model 5, the LMR and BLRT test results for the remaining models were statistically significant. Although Model 4 slightly outperformed Model 3 in terms of fit indices, its lower average posterior probabilities for individual class assignments and limited sample size reduced its clinical interpretability and practical utility. In Model 3, the average posterior probabilities for each latent class were all above 90% (see Table 3), indicating high reliability in the classification results. Therefore, this study ultimately selected a three-class latent profile model to assess FCP among older adults with stroke.

**Table 2:** Latent class model fit comparison.

Model	AIC	BIC	aBIC	Entropy	LMR (*p*)	BLRT (*p*)	Probabilities of classes (%)
1	4470.908	4503.115	4477.729	—	—	—	—
2	3938.785	3991.121	3949.869	0.835	0.000	0.000	38.9/61.1
3	3710.544	3783.009	3725.891	0.828	0.005	0.000	17.8/45.2/37.0
4	3540.211	3676.806	3603.821	0.861	0.043	0.000	13.3/30.4/14.3/42.0
5	3540.025	3652.749	3563.898	0.892	0.149	0.000	13.6/29.2/41.3/1.2/14.7

aBIC, Samplesize adjusted Bayesian Information Criterion; AIC, Akaike Information Criterion; BIC, Bayesian Information Criterion; BLRP, Bootstrapped Likelihood Ratio Test; LMR, Lo-Mendell-Rubin Adjusted Likelihood Ratio Test.

**Table 3:** Average probability of three-profile model (*N* = 414, %).

Category	C1	C2	C3
C1	0.943	0.057	0.000
C2	0.027	0.906	0.067
C3	0.000	0.070	0.930

### 3.3 Naming latent categories of future care preparation

**Figure 1:**
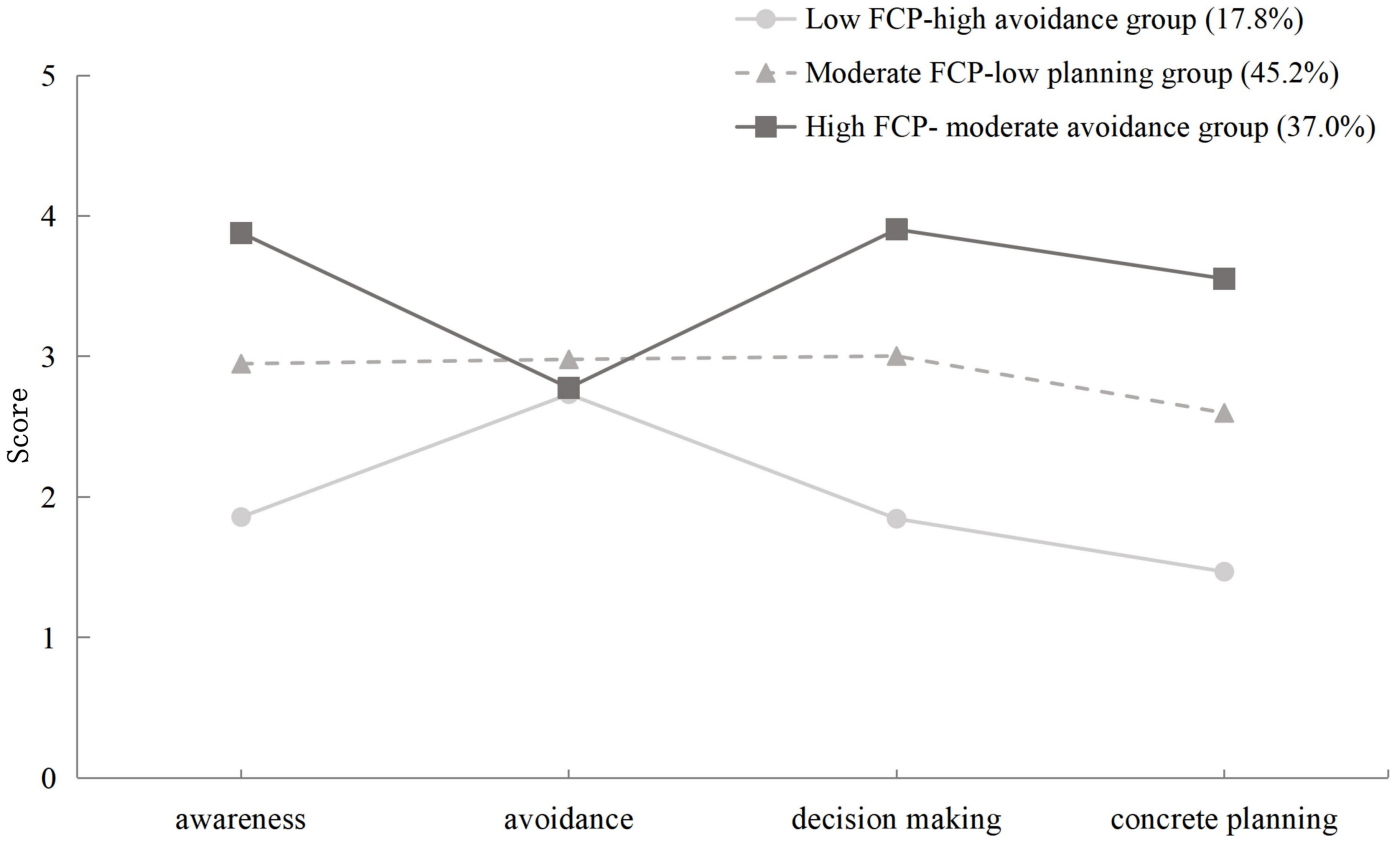
Latent profile plot based on the FCP for older adults with stroke.

Based on the results of latent profile analysis, this study created a profile plot (Figure 1) depicting the score distributions across the four dimensions of the PFCN-14 among three categories of older adults with stroke. In the plot, the horizontal axis represents each dimension of the scale, while the vertical axis indicates the mean item score for the corresponding dimension. By integrating the latent profile classifications with the score pattern characteristics depicted in the plot, these three latent categories were named accordingly.

Profile1 scored the lowest on the three positive preparation dimensions—awareness, decision-making, and concrete planning—indicating an overall low level of FCP. Additionally, it had the lowest score on the avoidance dimension among the three categories. Therefore, it was named the “low FCP-high avoidance group,” accounting for 17.8% of the sample. Profile2 demonstrated moderate scores across all four dimensions; however, its score on the concrete planning dimension was significantly lower than its performance on the other dimensions. Consequently, it was named the “moderate FCP-low planning group,” representing 45.2% of the sample. Profile3 scored the highest on the three positive preparation dimension—awareness, decision-making, and concrete planning. However, its score on the avoidance dimension fell between those of the first and second groups, indicating a moderate level of avoidance. Thus, it was named the “high FCP-moderate avoidance group,” comprising 37.0% of the sample.

### 3.4 Multiple logistic regression analysis of factors influencing latent categories of future care preparation

To explore the influencing factors of the latent categories of FCP among older adults with stroke, an unordered multivariate logistic regression analysis was conducted. The three latent categories—low FCP-high avoidance group, moderate FCP-low planning group, and high FCP-moderate avoidance group—were used as the dependent variables. Variables that were statistically significant in the univariate analysis were included as independent variables, with the low FCP-high avoidance group serving as the reference category. The assignment of independent variables is shown in Table 4. The regression analysis results revealed that scores on expectations of filial responsibility, age, gender, closeness with children, and living arrangements significantly influenced the latent categories of participants’ FCP (all *p* < 0.05), as presented in Table 5.

**Table 4:** Assignment of the independent variable.

Variable	Assignment
Age	60–69 = 1, 70–79 = 2, ≥80 = 3
Sex	Men = 1, women = 2
Educational level	Primary school/lower = 1, middle school = 2, high school = 3, junior college/higher = 4
Number of children	≤1 = 1, 2-3 = 2, ≥4 = 3
Closeness to children	Yes = 1, No = 2
Sex of children	Men = 1, women = 2, both = 3
Place of residence	City = 1, rural = 2
Living arrangements	Living alone, living with family, others
Filial responsibility expectations	Continuous variables are substituted with their measured values

**Table 5:** Multiple logistic regression analysis of latent categories of preparation for future care among the participants (*N* = 414).

Independent variable	Moderate FCP-low planning group					High FCP-moderate avoidance group				
	*β*	SE	Wald *χ*^2^	*p*	OR (95% CI)	*β*	SE	Wald *χ*^2^	*p*	OR (95% CI)
Filial responsibility expectations	−0.149	0.031	22.510	<0.001	0.862 (0.811, 0.916)	−0.226	0.035	41.397	<0.001	0.797 (0.744, 0.854)
Age										
60–69	1.787	0.563	10.089	0.001	5.971 (1.982, 17.985)	1.714	0.672	6.505	0.011	5.551 (1.487, 20.719)
70–79	0.893	0.456	3.846	0.049	2.443 (1.001, 5.971)	1.280	0.575	4.957	0.026	3.596 (1.166, 11.096)
Sex										
Men	−0.436	0.359	1.469	0.226	0.647 (0.320, 1.308)	−0.862	0.402	4.597	0.032	0.422 (0.192, 0.929)
Closeness to children										
Yes	1.069	0.344	9.666	0.002	2.912 (1.484, 5.713)	2.898	0.452	41.141	<0.001	18.144 (7.483, 43.991)
Living arrangements										
Living alone	1.012	0.702	2.081	0.149	2.751 (0.696, 10.881)	−0.610	0.816	0.560	0.454	0.543 (0.110, 2.688)
Living with family	1.089	0.534	4.169	0.041	2.972 (1.045, 8.458)	0.137	0.550	0.062	0.804	1.147 (0.390, 3.373)

Specifically, compared to the low FCP-high avoidance group, older adults with stroke with lower expectations of filial responsibility (OR = 0.862, 95% CI = 0.811–0.916), aged between 60–79 years (OR = 5.971, 95% CI = 1.982–17.985; OR = 2.443, 95% CI = 1.001–5.971 for different age subgroups within this range), having a close relationship with their children (OR = 2.912, 95% CI = 1.484–5.713), and living with family members (OR = 2.972, 95% CI = 1.045–8.458) were more likely to belong to the moderate FCP-low planning group. In contrast, compared to the low FCP-high avoidance group, patients with lower expectations of filial responsibility (OR = 0.797, 95% CI = 0.744–0.854), aged between 60–79 years (OR = 5.551, 95% CI = 1.487–20.719; OR = 3.596, 95% CI = 1.166–11.096 for different age subgroups within this range), female (OR = 0.422, 95% CI = 0.192–0.929), and having a close relationship with their children (OR = 18.144, 95% CI = 7.483–43.991) were more likely to be classified into the high FCP-moderate avoidance group.

## 4 Discussion

### 4.1 Profiles of future care preparation in older adults with stroke

The results of this study indicate that the total score for FCP among 414 older adults with stroke was (41.37 ± 9.47) points, which is higher than the score of (39.08 ± 12.02) points previously reported in a survey of community-dwelling older adults (6). This finding suggests that stroke, as a definitive health crisis, may have heightened patients’ awareness and preparedness regarding their future care needs. Therefore, hospitalization provides an ideal window for education on FCP. Clinicians should utilize this opportunity to provide structured guidance and support, helping patients begin to consider their FCP beyond the stable phase of the disease.

However, patients may exhibit distinct psychological and behavioral patterns in their engagement with FCP, necessitating differentiated intervention strategies. To explore this heterogeneity, we conducted a latent profile analysis and identified three distinct FCP profiles among older adults with stroke: the low FCP-high avoidance group (17.8%), the moderate FCP-low planning group (45.2%), and the high FCP-moderate avoidance group (37.0%).

The low FCP-high avoidance group scored the lowest on the three positive preparation dimensions—awareness, decision-making, and concrete planning—indicating insufficient awareness and cognitive engagement with FCP, a lack of clear care preferences, and the absence of specific plans. Concurrently, this group also obtained the lowest score on the avoidance dimension, suggesting a pronounced tendency to avoid thoughts or discussions related to FCP. Sociodemographically, this group mainly consisted of patients over 80 years old, living alone or with non-family members, residing in rural areas, not closely connected to their children, and displaying the highest filial piety scores. They may avoid future risk thinking due to old age, weak social support, and strong filial piety, prioritizing current emotional health over active preparation (32). Healthcare workers should identify them, guide through family interviews, and family members should offer support without full takeover to reduce their burden and avoidance.

The moderate FCP-low planning group comprised 45.2% of participants. They scored moderately on awareness and decision-making, indicating initial recognition of future care needs and some care preferences. However, their concrete planning scores were significantly lower, reflecting a failure to translate intentions into actionable plans. Notably, they scored highest on the avoidance dimension, meaning they were most open to discussing FCP. This “low avoidance yet low planning” pattern suggests the core obstacle is not FCP avoidance but limited capacity to move from intention to execution. Demographically, this group mainly consisted of married men aged 70–79, living with family and close to their children, yet with relatively low education. Despite strong family support and open attitudes, limited health literacy and planning skills, possibly compounded by post-stroke executive dysfunction, may hinder them from concretizing care arrangements (33).

The high FCP-moderate avoidance group scored highest across the three positive preparation dimensions—awareness, decision-making, and concrete planning—indicating that these patients had a clear understanding of their future care needs and were able to make decisions and formulate specific plans. Their scores on the avoidance dimension were moderate, suggesting that while they could generally confront future care-related topics, they still retained some degree of psychological defensiveness. This implies that active FCP engagement and moderate avoidance tendencies may coexist; even the most prepared patients could not entirely eliminate concerns about potential disability or dependence following a stroke. Demographically, this group was predominantly aged 60–69, mostly female, and generally had higher educational levels, close relationships with their children, and urban residency. These characteristics suggest that relatively younger female patients with strong social support and educational resources not only possessed greater planning capacity and motivation but also demonstrated higher psychological resilience, enabling them to better accept future uncertainties and take proactive preparatory actions (34).

Notably, the three latent profiles exhibited the most pronounced differences in the avoidance dimension. As an acute, sudden-onset event with disabling potential, stroke introduces uncertainty in rehabilitation trajectories, which may heighten patients’ psychological defensiveness regarding FCP (35). The low FCP-high avoidance group, while maintaining short-term emotional stability through strong avoidance, ultimately missed opportunities for preparation. In contrast, the moderate avoidance observed in the high FCP-moderate avoidance group may serve as a protective adaptive mechanism, as fully confronting disability could induce anxiety and disrupt recovery (36). Although the moderate FCP-low planning group showed the lowest avoidance tendency, their limited planning capacity prevented them from translating openness into concrete actions. These findings suggest that clinical interventions should adopt tailored strategies based on different levels of avoidance.

### 4.2 Association between demographic factors and future care preparation

The results of this study indicate that older adults with stroke aged 60–69 are more likely to be classified into the high FCP-moderate avoidance group, while those aged 70–79 tend to fall into the moderate FCP-low planning group. This suggests that among the study population, advancing age is not accompanied by an increase in the level of FCP; instead, it shows a certain declining trend. This finding diverges from some previous studies, a discrepancy that may be attributed to differences in sample characteristics and health status (37, 38). Prior research has largely focused on community-dwelling older adults, whereas the present study examined older stroke patients with poorer health. Older patients may experience a marked decline in health status post-stroke, compounded by disease-related cognitive impairment, which diminishes their perceived control over their own lives (39). Consequently, they tend to focus more on current rehabilitation issues, thereby avoiding long-term planning or relying entirely on others for care (40). Compared with younger stroke patients, older adults face the combined effects of age-related functional decline and post-stroke sequelae, further undermining their capacity and motivation for FCP (41). Meanwhile, relatively younger older patients often exhibit greater awareness of their own health risks and have accumulated some experience in caring for others, which provides them with stronger motivation and cognitive foundation to advance FCP (42). Therefore, healthcare professionals should pay particular attention to older patients with weaker preparation intentions, employing guiding strategies to break down FCP into actionable steps, thereby helping them overcome avoidance tendencies and establish effective coping preparations before further health deterioration occurs.

Additionally, compared to the low FCP-high avoidance group, female older adults with stroke are more likely to belong to the high FCP-moderate avoidance group. This was primarily attributed to the traditional gender roles in which women often assume greater responsibility for health management and caregiving within the family, rendering them more sensitive to health risks and more inclined to engage in advance preparation to avoid becoming a burden to their families (43). Proactive coping theory posits that taking preparatory measures in anticipation of potential stressors can effectively prevent or mitigate their impact (8). The caregiving experience accumulated by women in their daily roles provides them with a cognitive foundation and preparatory strategies for addressing future care needs. For stroke patients, female patients may draw upon their prior caregiving experiences to formulate more specific expectations and pragmatic plans regarding the care needs likely to arise after stroke (44).

### 4.3 Influence of family and cultural factors on future care preparation

Our study found that the closeness of the relationship with children is a significant influencing factor on FCP among older adults with stroke. As the main workforce in society, adult children often reduce communication and companionship with their parents due to work burdens, which may weaken intergenerational connections and subsequently increase negative emotions and even the risk of death among older patients (45). Maxfield et al.’s (46) research reveals a significant negative correlation between negative emotions and the dimension of specific planning, indicating that older adults with more negative emotions are less likely to engage in FCP. In contrast, older patients with closer and more supportive intergenerational relationships tend to adopt health-promoting behaviors, enjoy better overall health, and are motivated to prepare for future care (47, 48). Older adults with stroke living with family members are more likely to belong to the moderate FCP-low planning group. When older patients live with their spouses and children, interactions and support from family members help reduce the risk of social isolation (49). The more social support older patients receive, the fewer negative views they have about future care planning and the higher their enthusiasm for preparation (37).

Intergenerational relationships are an important source of social support and emotional connection for older patients (48). However, contrary to Pan et al. (50), the present study found that excessively high expectations of filial responsibility reduced FCP levels in older adults with stroke. This may be because receiving emotional support from children diminished their perceived necessity of FCP (51). Particularly when post-stroke cognitive or psychological vulnerability induced avoidance tendencies, suppressed autonomous planning motivation, and even reduced receptivity to professional care. Functional recovery after stroke often requires long-term care, and close intergenerational relationships provided sustained emotional support and rehabilitation encouragement, alleviating depressive symptoms and isolation while enabling patients to begin contemplating FCP during disease stability (48). However, as the older stroke population grew, the availability of children to assume caregiving responsibilities decreased, creating a gap between care expectations and children’s actual support capacity (52). This gap potentially exacerbated family tension and caregiving burden, preventing stroke patients from receiving timely and professional rehabilitation support within family settings and thereby delaying functional recovery (53).

Based on Thomas’s (54) social identity theory, it was suggested that, on one hand, family members should improve the physical and mental health status of older adults through active and effective intergenerational communication, helping older adults with stroke clarify their personal preferences before disease progression, thereby reducing potential future medical decision-making conflicts and promoting the effective implementation of FCP. On the other hand, healthcare professionals should guide families of older adults with stroke to transform traditional filial responsibility into an open and collaborative planning framework, and enhance patients’ self-efficacy and capacity for FCP by strengthening their sense of self-worth and social role identity.

### 4.4 Implications for clinical practice and family support

The findings of this study have important implications for clinical practice and family support. First, clinical interventions should adopt stratified strategies based on patients’ profile characteristics. For the low FCP-high avoidance group, healthcare providers should prioritize psychological counseling to reduce avoidance tendencies before gradually guiding patients to address future care issues. For the moderate FCP-low planning group, structured planning tools and skills training should be provided to help translate openness into concrete actions. For the high FCP-moderate avoidance group, planning details should be refined while affirming their preparation awareness. Second, family members should convey supportive attitudes that assist preparation rather than assume full responsibility. Open intergenerational communication helps patients express concerns and clarify care preferences. Transforming traditional filial responsibility into a collaborative family planning model enhances both family support and patients’ planning capacity while respecting patient autonomy. Finally, hospitalization represents a critical window when stroke patients’ health awareness is activated. It is recommended that medical institutions integrate FCP education into routine rehabilitation care, guiding patients to contemplate post-stability care issues during disease stabilization.

### 4.5 Limitations and future directions

This study has several limitations. First, due to the cross-sectional design, it is impossible to infer temporal or causal relationships among the variables. Second, the sample is solely sourced from a single province in China, and the generalizability of the findings is limited given the differences in economic, cultural, and medical conditions across regions. Third, the data in this study are primarily based on patients’ self-reports. In the context of Chinese culture, concerns about “face” may lead to social desirability bias, affecting the authenticity of the responses. Additionally, the measurement of FCP solely through questionnaires may be subject to common method bias. Future research should adopt a longitudinal design, incorporate multi-center samples from across the country to enhance generalizability, and integrate objective variables (such as clinical indicators or family assessments) to inform the construction of an evidence-based, individualized, and family-collaborative older adult care support system.

## 5 Conclusion

This study identified three latent profiles of FCP among older adults with stroke and demonstrated that younger age, female gender, closer intergenerational relationships, and lower expectations of filial responsibility were associated with higher levels of proactive FCP. Clinicians should focus on early identification of patients with low preparation and high avoidance, particularly older males living alone, and employ structured guidance strategies. Families should support autonomy of older adults through open and collaborative communication, mitigating the potential inhibiting effects of traditional filial piety norms.

## Data Availability

The raw data supporting the conclusions of this article will be made available by the authors, without undue reservation.
